# The Immunomodulatory Effects of Mesenchymal Stem Cell Polarization within the Tumor Microenvironment Niche

**DOI:** 10.1155/2017/4015039

**Published:** 2017-10-17

**Authors:** Cosette M. Rivera-Cruz, Joseph J. Shearer, Manoel Figueiredo Neto, Marxa L. Figueiredo

**Affiliations:** Department of Basic Medical Sciences, Purdue University College of Veterinary Medicine, West Lafayette, IN 47907, USA

## Abstract

Mesenchymal stem cells (MSCs) represent a promising tool for cell therapy, particularly for their antitumor effects. This cell population can be isolated from multiple tissue sources and also display an innate ability to home to areas of inflammation, such as tumors. Upon entry into the tumor microenvironment niche, MSCs promote or inhibit tumor progression by various mechanisms, largely through the release of soluble factors. These factors can be immunomodulatory by activating or inhibiting both the adaptive and innate immune responses. The mechanisms by which MSCs modulate the immune response are not well understood. Because of this, the relationship between MSCs and immune cells within the tumor microenvironment niche continues to be an active area of research in order to help explain the apparent contradictory findings currently available in the literature. The ongoing research aims to enhance the potential of MSCs in future therapeutic applications.

## 1. Introduction

The tumor microenvironment is composed of extracellular matrix and nontumor stromal cells (fibroblasts, endothelial, and immune cells). Communication between the tumor and stromal cells plays a pivotal role in the progression of cancer [1]. Mesenchymal stem/stromal cells (MSCs) [2] represent one population of cells that are found within the tumor stroma and have shown potential for either promoting or inhibiting tumor growth [3]. MSCs are often utilized for their therapeutic potential since they have the capacity to differentiate into nonhematopoietic cell lineages, promote tissue repair and regeneration, and modulate immune responses [4, 5]. Although MSCs isolated from the bone marrow (BM-MSCs) are the most commonly studied, MSC populations also can be obtained from many other tissue sources, including the placenta, skin, adipose tissue, and Wharton's jelly [6]. While the characteristics used to define these cells vary by laboratory, generally, MSCs share an ability to adhere to plastic *in vitro* and possess an immunophenotype that includes detectable expression of cluster of differentiation (CD) 105, CD73, and CD90 and negative expression of CD45, CD34, CD14, CD19, CD3, and human leukocyte antigen- (HLA-) DR surface markers [5–7]. In addition, MSCs are characterized by their capacity to differentiate at a minimum into adipogenic, chondrogenic, and osteogenic lineages *in vitro* [5, 8]. MSCs have emerged over the last decade as a promising modality for cell therapy, for applications ranging from regenerative medicine to tumor therapy.

Some of the general advantages of using MSCs for cell therapy include the ease of expansion and storage ex vivo and their ability to avoid immune clearance [9]. In addition, MSCs home to sites of injury, where they secrete extracellular matrix components, chemokines, and cytokines [10]. The secretome of these cells has functions associated with chemoattraction and modulation of immune cells, angiogenesis, and support of cellular growth and proliferation [11]. Because MSCs home towards tumors in a similar fashion as they home to injury sites, they can be useful for delivering cell-based therapeutics to tumor sites. The mechanisms utilized by MSCs to home towards tumors include several signaling axes, including stromal cell-derived factor (SDF-1 or CXCL12), its receptors, C-X-C-chemokine receptor type 4 (CXCR4), and related chemokine signals (CXCL10, CXCR3), as well as the immune regulatory cytokine transforming growth factor beta (TGF*β*) [10]. However, despite progress in understanding the nature and function of MSCs within the tumor microenvironment, many questions remain unanswered regarding their safety and efficacy for clinical use. This is due to the duality associated with MSC signaling once they reach the tumor microenvironment. For example, depending on the context and tumor type, MSCs have been found to either support or inhibit tumor progression [12].

**Figure 1:**
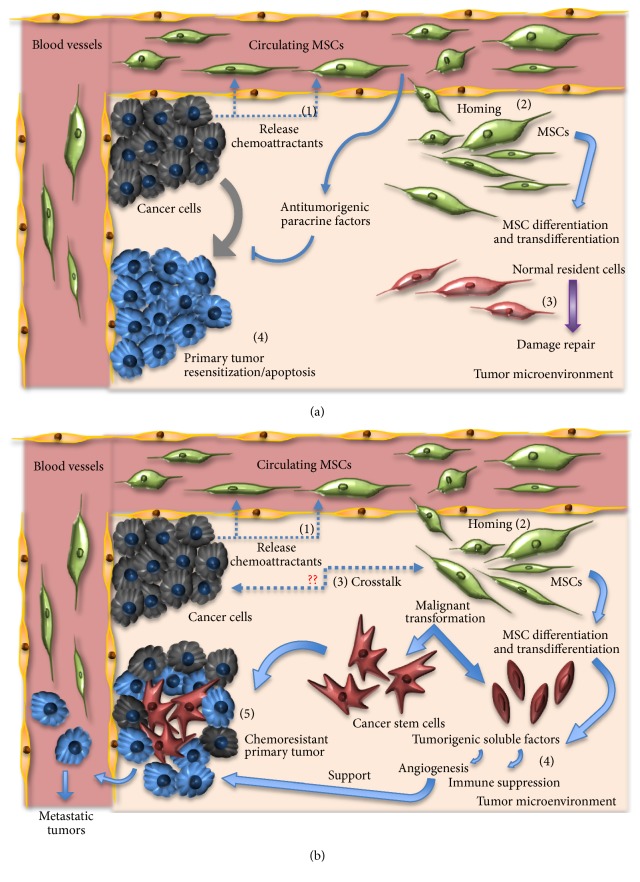
The role of MSCs in the tumor microenvironment. (a) The antitumor effects of MSC. Circulating MSC may release antitumor paracrine factors causing primary tumor resensitization and cancer cell apoptosis, while infiltrating MSCs may differentiate to contribute to tissue repair. MSCs arrive at tumors following chemoattraction (1), home towards tumors (2), with the goal of performing damage repair (3), and induce primary tumor resensitization and apoptosis (4). (b) The protumorigenic effects of MSCs. Infiltrating MSCs are attracted to tumors via chemoattractants (1), home to tumors (2), participate in secretory crosstalk with tumor cells (3), release proangiogenic and immune-suppressive soluble factors (4), and may support the growth of chemoresistant tumors (5).

MSCs elicit many of these effects on other cells through the release of paracrine factors, which can cause primary tumor resensitization and cancer cell apoptosis (Figure 1(a)). MSCs that infiltrate tumors come from local or distant sources [13] and may differentiate and/or transdifferentiate into normal resident cells in an attempt to contribute to tissue repair (Figure 1(a)). Within the tumor microenvironment, MSCs are able to induce cancer cell survival, stemness, and chemoresistance following their differentiation into cancer-associated fibroblasts (CAF) and by their release of soluble factors favoring angiogenesis and immune suppression (Figure 1(b)). Once MSCs have infiltrated the tumor microenvironment, the presence of tumor necrosis factor (TNF), interleukin- (IL-) 1, and interferon *γ* (IFN*γ*) or hypoxic conditions all stimulate MSCs to release proangiogenic and immunosuppressive factors including epidermal growth factor (EGF), platelet-derived growth factor (PDGF), fibroblast growth factor (FGF), vascular endothelial growth factor (VEGF), and IL-6 and IL-8 [14]. Some of these paracrine factors released by MSCs such as IL-10 and TGF*β* attract immune cells locally, where MSCs can inhibit their activation and proliferation [15]. The combination of angiogenic and immunosuppressive factors allows for tumors to escape the immune surveillance, proliferate, and metastasize [16]. However, contradictory evidence has been shown also regarding the ability of MSCs to inhibit tumor growth.

The discrepancies surrounding the ability of MSCs to either promote or inhibit tumor progression include factors such as tissue source, individual secretomes, nature of interactions with cancer cells and immune cells, type of cancer or cancer cell lines, and experimental conditions [10, 12]. Additionally, several studies are designed with MSCs from healthy donors which are functionally different from MSCs from cancer patients which likely undergo cellular and molecular changes in direct or indirect (secretome) interactions with cells in the tumor stroma [12, 17]. Therefore, there needs to be a much better understanding of the mechanism(s) regarding the communication between MSCs and immune cells within the tumor microenvironment and how that might impact tumor progression. Gaining a better understanding of these factors might allow clinicians to harness the MSC secretory phenotype in order to optimize their therapeutic potential against cancer.

## 2. Mesenchymal Stem/Stromal Cells and Their Microenvironment

**Figure 2:**
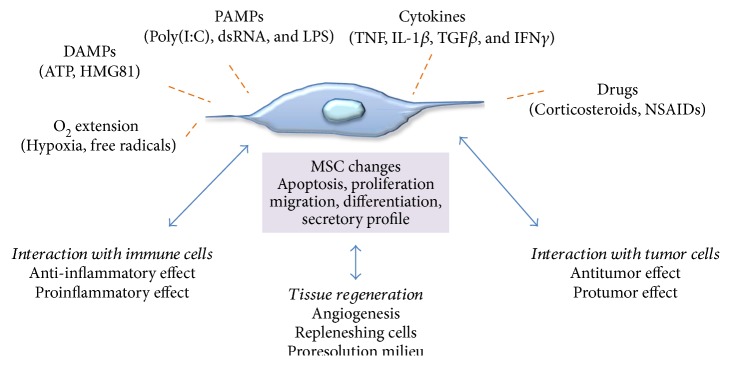
Interactions of MSCs with the microenvironment. Summary of environmental factors that can influence cellular responses of MSCs in the tumor microenvironment and/or tissue regeneration settings.

In recent years, as cellular therapy using MSCs has become a therapeutic option to treat numerous diseases, several studies have examined the role of the microenvironment on MSC biology. When MSCs are introduced into a pathological milieu, then, they can exhibit increased or reduced survival and can alter their differentiation or immunomodulatory characteristics based on the physical and biochemical features of the microenvironment encountered. The response of MSCs to environmental cues might alter their phenotype towards proinflammatory or anti-inflammatory activities, depending on the context, and this duality of function has been compared to the polarization observed in macrophages. It is well established that macrophages may become either proinflammatory or anti-inflammatory depending on the cytokine environment to which they are exposed [18]. IFN*γ* plus LPS promotes predominantly M1 or proinflammatory macrophages with a relative increase in TNF*α* production and a reduction in IL-10 secretion. IL-4, on the contrary (alone or with IL-10 and TGF*β*), promotes M2 macrophages, with a prominent anti-inflammatory phenotype with marked IL-10 release. This has led investigators to pursue the concept that MSCs, similar to macrophages, can be rendered either proinflammatory or anti-inflammatory depending on the cues they receive from their microenvironment. And this plasticity may help to explain their ability to be both pro- and antitumorigenic. Several environmental stimuli can impact how MSCs alter their apoptotic, proliferative, migratory, differentiation, and secretory profiles (Figure 2), and these will be detailed in the following section.

### 2.1. Hypoxia

Oxygen tension within a tumor is highly heterogeneous and can be found present at levels almost as low as anoxic conditions (close to no oxygen). Increasing hypoxia (low oxygen) within a tumor could be an indicator of tumor progression and can lead to the selection of highly invasive cancer cells with greater resistance to therapies. Differences in the oxygen tension levels in the tumor microenvironment have been associated with the modulation of properties of tumor stromal components [19]. Often, MSCs are usually *in vitro* in normoxic conditions, a higher oxygen tension level than what would be found *in vivo* [20]. Studies suggest that differences in oxygen tensions can lead to changes in the commonly studied properties of MSCs such as stemness [21], differentiation [16, 22–24], and secretome [25].

For example, hypoxia can promote MSC proliferation, enhance their migration, and maintain their stemness [21–23, 26–31]. The yield of differentiation into nonhematopoietic lineages is also altered in these cells as a result of variable oxygen tensions. The osteogenic differentiation potential has been reported to be increased under hypoxic conditions when compared to that of normoxic-cultured MSCs [23, 29, 30]. Chondrogenic differentiation has been inhibited by culture under hypoxic conditions [25]. The adipogenic differentiation potential has been reported to be either enhanced [32] or inhibited [25, 29], and the difference in findings could be attributed to differences in MSC sources, as well as to differences in experimental conditions.

In laboratory settings, a wide variety of experimental protocols is used to study the effect of low oxygen tensions on MSC behavior, leading to difficulties in the comparison of the data. However, as indicated by Buravkova et al., the available data can be divided into two main groups utilizing duration of exposure as a common ground: MSCs grown under normoxic conditions and later exposed to acute short-term hypoxia (up to 72 hrs) and MSCs cultured permanently under hypoxic conditions [26]. From this perspective, the effects of hypoxia on MSC properties seem to occur in two phases; a short-term acute treatment under hypoxic conditions causes cell damage involving apoptosis, followed by adaptation mechanisms including a switch to an anaerobic glycolysis metabolism [26, 27] and maintenance of an undifferentiated multipotent state [26].

Paradoxically, pointing to a dual role for MSCs, some studies suggest that hypoxia pretreatment can promote more efficient cartilage repair [24], likely through induction of hypoxia-inducible factor 1 alpha. Likewise, umbilical cord-derived MSCs preconditioned with hypoxia can be more efficient in treating mouse hind limb ischemia [33]. Hypoxia may induce a significant increase in triglycerides, fatty acids, and diacylglycerols in MSCs, whereas blocking hypoxia might enhance production of angiogenic factors like VEGF and angiopoietin-2 [34]. Hypoxic conditions might also enhance the supportive role of MSCs on endothelial progenitors, for example, in diabetic rats with hind limb ischemia [35], which might be detrimental in the context of tumors, for example. However, the consequence of changes in oxygen tensions within the tumor microenvironment in the context of MSC interactions is not yet well understood. It is known that several soluble molecules are upregulated by MSCs in response to hypoxia, including cell cycle-regulating proteins such as VEGF and IGF-II [23, 28] and hypoxia-inducible factors (HIFs) with roles in the promotion of macrophage recruitment, primary tumor growth [32], and metastasis of breast cancer [32, 36] and induction of proangiogenic and chemotactic secretion factors such as MCP-1, IL-8, and RANTES [25]. The altered MSC secretome would likely impact promotion of tumor growth and alter infiltration of immune cells.

### 2.2. MSCs and Cytokines

When exposed to TNF*α*, a major proinflammatory cytokine, MSCs display reduced proliferation and caspase-dependent apoptotic pathways are activated through p38 mitogen-activated protein kinase (MAPK) and c-Jun N-terminal protein kinase (JNK) pathways [21]. Conversely, TNF*α* can stimulate MSC migration and ICAM-1 expression, a molecule important in transendothelial migration of MSC [20]. TNF*α* pretreatment also can induce secretion of proangiogenic growth factors including fibroblast growth factor (FGF) 2, VEGF, or IL-8, promoting microvessel formation [36], but reduces the ability of MSCs to block neutrophil influx and improve perfusion of the jejunum in another model [37]. Interestingly, TNF*α* also can modulate anti-inflammatory effects on MSCs, enhancing prostaglandin E2 (PGE2) release and promoting IL-10-expressing anti-inflammatory macrophages [38]. Moreover, TNF*α* can induce the release of a potent anti-inflammatory protein called TNF*α*-stimulated gene 6 or TSG-6 [39].

Another example of a cytokine with effects on MSCs is interferon gamma or IFN*γ*. MSCs get “licensed” with T cell inhibitory properties when this cytokine is produced by CD4^+^ helper T cells and cytotoxic CD8 T lymphocytes. There seems also to be some species-specific changes in MSCs in response to IFN*γ*, leading to upregulation of IDO in human MSCs and upregulation of iNOS in murine MSCs. The net result is that IDO ultimately depletes tryptophan in the local microenvironment, rendering lymphocytes inactive and unable to proliferate, while iNOS increases local NO concentration, leading to inactivation of several proinflammatory genes in lymphocytes [40]. IFN*γ* also upregulates ICAM1 and VCAM1 in MSCs [41], assisting these cells in contacting lymphocytes and other immune cells for a maximized effect. MSCs treated with IFN*γ* also can express inhibitory costimulatory molecules such as B7 family coregulatory molecules B7-H1 [42], which can interact with CD4^+^ lymphocytes and block cell proliferation, promoting T cell anergy. Interestingly, there appears to be a critical threshold of an IFN*γ* concentration that can activate the immunosuppressive MSC effects [43], a threshold that might help explain differences among studies, even though differences in donor sources or culture conditions also may play a role in modifying the impact of IFN*γ* on MSCs. IFN*γ* can induce MHC classes I and II on MSCs, rendering them immunogenic [44]. Finally, another cytokine shown to block the anti-inflammatory properties of MSCs by reducing iNOS expression is TGF*β* [45], pointing to a feedback mechanism in the microenvironment whereby TGF*β* induction might promote the resolution of inflammation and/or tissue regeneration.

### 2.3. MSCs and Damage-Associated Molecular Pattern Molecules (DAMPs) or Pathogen-Associated Molecular Patterns (PAMPs)

DAMPs include nuclear high-mobility group box 1 protein (HMGB1), a chromatin-associated protein; cytosolic proteins such as S100 and purine metabolites (adenosine triphosphate, uric acid); and extracellular matrix (ECM) elements such as hyaluronic acid fragments. These proteins and ECM components are typically released into the microenvironment following tissue damage due to inflammation or other physical, chemical, or biological insults [46]. These molecules are sensed through binding to either Toll-like receptors TLR2 and 4 or receptors for advanced glycosylation products by MSCs, augmenting in many cases the trafficking and proliferation of MSCs. HMGB1 also can modulate the expression of IDO by MSCs, an immunomodulatory enzyme. Additionally, uric acid and S100A4 can act as chemoattractants of MSCs and enhance their immunomodulation effects by stimulating the expression of IL-10 and IDO in immune-suppressive lymphocytes [47]. For PAMPs, molecules produced by invading microorganisms, the detection by MSCs can occur via cytosolic membrane-bound pattern recognition receptors (PRR) [46]. TLRs are a subset of these PRR and are abundant in several immune and epithelial cells. TLRs will be discussed in more detail in Section 3 of this review.

### 2.4. Mesenchymal Stem/Stromal Cells and Drugs

It is important to also understand the effect that drugs can have on MSCs, since cells infused during regenerative therapies might be coadministered with agents such as corticosteroids or nonsteroidal anti-inflammatory drugs. The immunomodulatory properties of MSCs depend on their production of sufficient amounts of prostaglandins (PGE2) [48]. Coadministration with NSAIDs, for example, might reduce the effects of PGE2 and modify the effects of MSCs at sites of inflammation. On the other hand, tumor therapy applications might use gene-modified MSCs in coadministration with chemotherapeutics or immunotherapies to enhance their effects. MSCs loaded with chemotherapeutics can be used as delivery vehicles to tumors, as cultured MSCs are capable of selectively homing to and surviving in a variety of preestablished solid tumors (breast, colon, melanoma, and others) while being excluded from normal tissues [49]. The safe use of MSCs to treat cancer or noncancer diseases in patients that have undiagnosed, early-stage cancer requires understanding the fate and functions of MSCs and their interactions with tumors. Interestingly, instead of gene-modified MSCs, incorporation of drug-laden nano/microparticles inside the cell or on the cell surface also can be done [49].

The next sections of this review will focus on the changes MSCs impart onto the tumor microenvironment that relate to immune cell changes and also on the mechanisms of MSC polarization, which may help explain the dual antitumorigenic and protumorigenic nature of these cells as they interact and modulate various effects on tumor cells.

## 3. The Interaction of MSCs and the Immune System in the Tumor Microenvironment

The primary role of the immune system is to defend the body against the external environment and pathogens. The induction of specific immune responses, such as the production of antibodies to a particular pathogen, is known as an adaptive or acquired immune response and is typically acquired during the lifetime of an individual as an adaptive response to a specific pathogen. This distinguishes such responses from innate immunity, which is a type of inborn defense in that its action does not depend upon prior exposure to a particular pathogen. Innate immunity and adaptive immunity are orchestrated by sets of interacting but distinct cell types, and both responses are found to be important in tumor elimination or relapse, depending on the context. MSCs can impact both innate and adaptive immune responses within tumor of various types.

### 3.1. Innate Immunity Changes Mediated by MSCs in the Tumor Microenvironment

Without a properly functioning innate immune system, aberrant cell populations run the risk of going unchecked within an immunosuppressive environment conducive for the progression of cancer. Cell types of the innate immune system, including macrophages, natural killer (NK) cells, and dendritic cells (DC), possess mechanisms optimized for the detection and removal of tumor cells. The coordinated activation and response of the innate immune system are quite complex, involving the recruitment and maturation of a wide range of cell types. The inflammatory tumor microenvironment plays a key role in the recruitment of many of these innate immune cells through the release of proinflammatory cytokines [50]. Upon recruitment, these cells are then further differentiated in order to properly carry out their designated functions to restore a homeostatic microenvironment. The initiation of the proinflammatory signal may depend on local expression of IFN*β* by DC to promote initial innate recognition of tumors [51]. Infectious disease models have indicated at least three pathways of innate immune sensing that can drive IFN*β* upregulation at the transcription level. These are Toll-like receptor (TLR) signaling, retinoic acid-inducible gene 1 (RIG-I) sensing of cytosolic RNA, and the stimulator of interferon genes (STING) pathway sensing of cytosolic DNA from dying tumor cells [26]. In the case of tumors, the same mechanisms can be utilized to activate the innate system by “sterile immunity” with the participation of these innate immune sensing pathways, which involve stress-associated or damage-associated molecular patterns triggering innate immune activation.

**Figure 3:**
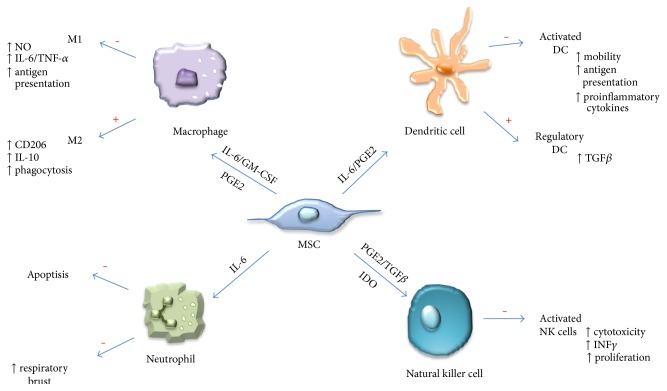
Interactions of MSCs with innate immune cells. MSCs utilize diverse molecular mechanisms to suppress or promote innate immune cells. MSCs suppress macrophage polarization to M1, although they favor M2 polarization. MSCs inhibit NK cell and DC activation, differentiation, and effector functions. MSC-derived PGE2 contributes to all of these effects. MSC-produced IL-6 suppresses neutrophil apoptosis and respiratory burst and also contributes to inhibiting DC function. In the presence of IL-6 and GM-CSF, MSCs also can affect macrophage function, while TGF*β* and IDO suppress NK cell function. In addition, MSCs also favor the generation of regulatory DCs. Effects of MSC: + indicates function promotion and − indicates function suppression.

Emerging mechanisms associated with infiltrations of innate immune cells into tumors have shown an important functional role for another component of the tumor microenvironment—stromal cells. The supportive stromal cell populations contribute their own cytokines that impact the innate immune response. MSCs are one of the stromal cell populations being actively studied in order to elucidate the effects they have on innate immune cell recruitment and functionality, due to their ability to influence multiple types of innate immune cells (Figure 3) [52]. Here, we highlight a few of the mechanisms in which MSCs influence the specific cell populations of the innate immunity.

#### 3.1.1. Immune-Activating Effects of MSCs on Innate Immunity Cells within the Tumor Microenvironment

The literature has few reports on immune-activating effects of MSCs on the innate immunity within tumors. Some immune-activating effects might include the effects of MSCs in increasing the phagocytic ability of cocultured macrophages. One study showed that macrophages cocultured with MSCs expressed high levels of CD206 and IL-10 and low levels of IL-12, suggesting development of alternatively activated macrophages [53]. Although the cocultured macrophages also expressed high levels of IL-6 and low levels of TNF*α* compared to controls, functionally, they displayed a higher level of phagocytic activity. These MSC-educated macrophages might represent a unique type of alternatively activated macrophage with a potentially significant role in tissue repair. Whether this macrophage type can be promoted within the tumor microenvironment following interaction with MSCs is not known.

Although far less is known about the role of neutrophils in interaction with MSCs, some studies suggest that polarization of MSCs via TLR3 (but not so much TLR4) activation preserves viable and functional neutrophils by amplifying the antiapoptotic effects that resting BM-MSCs would normally exert on these cells [54]. In addition, TLR3- and TLR4-activated BM-MSCs enhance the function of neutrophils, and the mechanism appears to be via the MSC secretome, as there was an absence of cell contact during incubation. Neutralizing experiments with MSC from various tissue sources revealed that the biological effects exerted on neutrophils by TLR3-activated MSC were mediated by the combined action of IL-6, IFN*β*, and granulocyte macrophage colony-stimulating factor (GM-CSF), while those exerted by TLR4-activated MSC mostly depended on GM-CSF. MSC thus can sustain and amplify the functions of neutrophils in response to TLR3 and TLR4 triggering and may consequently contribute to inflammatory disorders. Another study determined that BM-MSCs can enhance the ability of neutrophils to phagocytize bacteria and to promote bacterial clearance in the peritoneum and blood [55]. The beneficial effects of MSCs could be reversed upon neutrophil depletion, demonstrating the importance of neutrophils for this MSC response in a model of sepsis. The role of any interactions between neutrophils and MSCs in the tumor microenvironment is unknown, but these studies would suggest that MSCs might be able to enhance the respiratory burst and other functions of neutrophils, which could act in a tumor-inhibitory manner.

#### 3.1.2. Immune-Suppressive Effects of MSC on Innate Immunity Cells within the Tumor Microenvironment

The majority of the effects reported in the literature for the interaction of MSCs with innate immunity cells are of an immune-suppressive nature and involve several cell types, including macrophages, natural killer cells, and dendritic cells. Macrophages represent a major cell population involved in the innate immune response. The importance of macrophages and immunosurveillance of tumors has been well established [56]. However, these cells can have drastically different functions depending on their polarization state. In fact, macrophages can elicit anti-inflammatory (M1) or inflammatory (M2) properties following exposure to different polarization signals [18]. Many of these polarization signals come from cytokines released within the niche of the tumor microenvironment. Their recruitment and maturation are tightly regulated to ensure proper control and remove aberrant cell populations.

**Figure 4:**
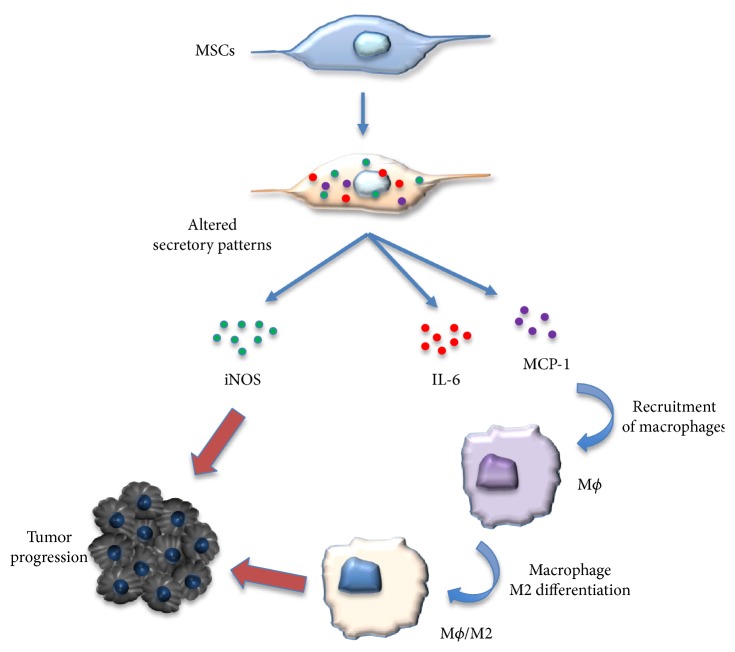
Interaction between macrophages and the MSCs in promoting tumor growth. M1 macrophages could activate the MSCs to adopt a regulatory phenotype, and the MSCs with an altered secretory profile promoted tumor growth by iNOS and MCP1 and induced macrophages toward M2-like macrophages.

MSCs actively influence the function of macrophages by influencing their polarization status (Figure 4) [57]. For example, MSCs shift the polarization of macrophages from a TNF*α*-secreting M1 signature to an immunosuppressive IL-10-expressing phenotype which may be mediated through a prostaglandin- (PGE-) 2-based mechanism [58]. Wolfe et al. also demonstrated that conditioned media collected from MSCs were able to induce an M2 phenotype in macrophages indicative of an upregulation of arginase 1 and CD206 [59]. This shift was also accompanied by an increase in IL-6 production by MSCs following coculture with M2 macrophages. While the duality of IL-6 expression is still under investigation, its expression has been associated with a range of protumorigenic functions including increased proliferation, angiogenesis, and immunosuppression [60]. Furthermore, MSCs isolated from neoplastic pancreatic tissue polarized macrophages into an M2 phenotype to a greater extent than “normal” or naïve MSCs [61]. This suggests that MSCs isolated from tumors change in response to the tumor microenvironment. Collectively, these studies suggest that the presence of MSCs can influence the fate of macrophages, which may alter their ability to detect and eliminate cancer cells, and this would create a more immunosuppressive environment.

Natural killer (NK) cells are a lymphatic cell population involved with the innate immune response. They play an essential role in the detection of cancer by discriminating healthy from unhealthy cell populations in order to properly mitigate a cytotoxic immune response [62]. The differential expression of CD56 has suggested that NK cells are present as a heterogeneous cell population within tumors, with distinct cytokine profiles and cytotoxic potential [63]. And similar to macrophages, MSCs can influence NK cell function through a range of processes. For instance, MSCs from umbilical cord, bone marrow, and adipose tissues have shown immunomodulatory effects by inhibiting activation of the CD56^low^ subset of NK cells, which was accompanied by a decrease in TNF*α* expression [64]. MSCs also were able to decrease the proliferation and NK cell function by increasing the expression of the suppressor of cytokine signaling 3 in a recent model of sepsis [65]. MSCs isolated from acute myeloid leukemia and lung cancer tissues or cultured in the presence of conditioned media from HeLa cells showed an increase in TLR4 expression compared to naive MSCs. This resulted in decreased cytotoxic function of NK cells potentially through the decreased release of the proinflammatory cytokines IL-6 and IL-8 by these MSCs compared to naïve BM-MSCs [66].

Interestingly, MSCs isolated from cancer patients do not always attenuate the function of NK cells. In fact, MSCs isolated from acute lymphoblastic leukemia patients increased the cytotoxic functionality of NK cells to a greater extent than healthy MSCs [67]. The influence of MSCs on the recruitment of NK cells has also been considered a potential therapeutic strategy. Genetically manipulated MSCs overexpressing sirtuin 1 have been shown to decrease tumor size in a subcutaneous tumor mouse model partly through the recruitment of NK cells [68].

Dendritic cells (DCs) play an important role in maintaining the activation of both the innate and adaptive immune response partially by being an important antigen-presenting cell (APC) type [69]. MSCs have shown the ability to alter the function of DCs. MSCs are able to suppress the maturation of DCs through the secretion of IL-10 and through activation of the signal transducer and activator of transcription (STAT)3 signaling, promoting decreased IL-12 production by DCs [70]. MSCs isolated from placental tissue were shown to attenuate the maturation process of human DCs as well as to alter the DC secretome by decreasing the secretion of IL-12 and IL-23 [71]. Within the tumor microenvironment, MSCs suppressed the ability of DC-mediated T cell mechanisms including IFN*γ* secretion and tumor cytotoxicity by reducing the amount of available cysteine excretion through a STAT3 mechanism [72]. MSCs isolated from chronic myeloid leukemia patients induced secretion of higher levels of TGF*β* from DCs which in turn increased the differentiation of regulatory T (Treg) cell populations [73]. While still an active area of research, Tregs have been shown to be important in immunosuppression and have been linked to cancer progression [74].

The maturation and recruitment of cells associated with the innate immune response are tightly regulated by cell-to-cell and paracrine communication within the tumor microenvironment. MSCs represent a biologically active stromal population that has been shown to influence these processes in a range of innate cell populations, highlighting the importance MSCs have in the regulation of the innate immune response even when not directly involved in the immune response.

### 3.2. Changes in the Adaptive Immunity Response Mediated by MSC in the Tumor Microenvironment

**Figure 5:**
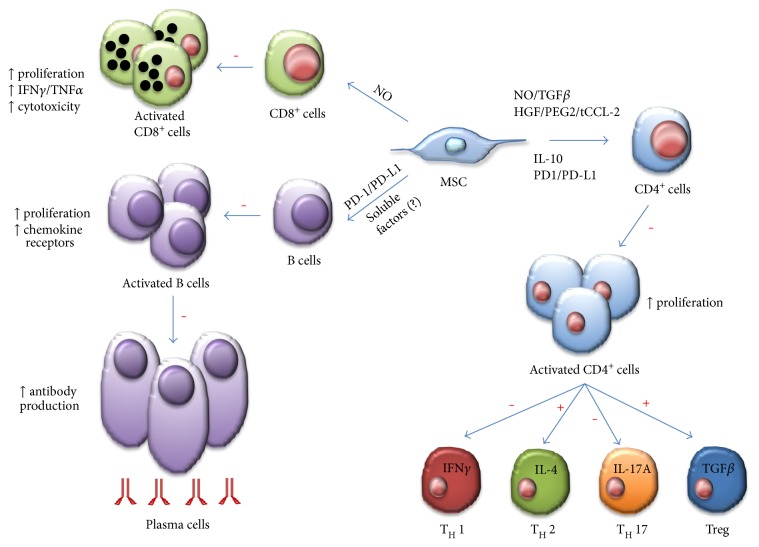
MSC immune suppression or promotion of adaptive immune cells. MSCs inhibit several aspects of B cell activity, including activation, proliferation, chemokine receptor expression, and differentiation to becoming antibody-secreting plasma cells. Unknown soluble factors and PD-1/PD-L1 ligation mediate these effects of MSCs on B cells. MSC can induce NO in response to inflammatory cytokine detection to suppress CD8^+^ T cell proliferation, cytokine production, and cytotoxicity. In response to activation by certain cytokines, CD4^+^ T cells can differentiate into numerous effector populations. MSCs produce soluble factors (NO, TGF*β*, HGF, PGE2, truncated CCL-2, and IL-10) and membrane-bound molecules (PD-1 ligation) to achieve suppression of CD4^+^ T cell proliferation and the polarization of CD4^+^ T cells towards TH1 and TH17 cells. MSCs also favor the development of TH2 and anti-inflammatory Treg populations. Effects of MSC: + indicates promotion and − indicates suppression.

Tumors are antigenic, and some display hundreds of mutations in coding exons, representing a large repertoire of antigens as potential targets for immune system recognition. But despite expression of abundant antigens, most cancers progress and evade destruction by the immune system. Analysis of the tumor microenvironment in patients with a variety of solid tumors has revealed that a major subset of tumors shows evidence of a CD8 T cell-infiltrated phenotype, but these become functionally inhibited by several mechanisms. These include programmed death ligand 1, expressed on tumor cells to limit activated T cell development and response; indoleamine-2,3-dioxygenase (IDO); and FoxP3^+^ Treg cells. The development of this phenotype appears, in part, to be promoted by type I interferon signaling and DCs. Another interaction that appears to have a role in driving adaptive immune cell infiltration and persistence is the presence of stromal cells, such as MSCs, in the tumor microenvironment. MSCs impact adaptive immune cell recruitment and phenotypes in different ways, by promoting either immune activation or immune suppression within the tumor microenvironment (Figure 5).

#### 3.2.1. Immune-Activating Effects of MSCs on Adaptive Immunity Cells within the Tumor Microenvironment

Immune activation by MSCs stems from the ability of these cells to activate allogeneic T cells in mixed leukocyte reactions (MLR) [4], an assay that assesses how T cell populations react to external stimuli by activation and proliferation. In a similar coculture model, MSCs have the ability to stimulate resting T cells to become activated and to proliferate [75]. Also, MSCs can behave as conditional APC in syngeneic immune responses [76], whereby TLR-activated MSCs recruit and activate immune inflammatory cells, likely through the secretion of proinflammatory cytokines by MSCs [77]. The clinical implications of this immune-activating phenotype are unknown, and whether these observations can be extended to MSC derived from other tissues is unclear at this time.

A very interesting report showed that human adipose-derived MSCs (ASCs) could induce what the authors called an “explosive” T cell proliferation, effectively activating resting immune cells [75]. When cocultured with peripheral blood mononuclear cells (PBMC), ASCs upregulated IL-6, IL-8, TNF, FGF, and VEGF, as well as IDO, suggesting strong crosstalk between cell populations. Following removal of ASC from the coculture, PBMC showed a large increase in proliferation, with a 25-fold increase after 7 days. The proliferating fraction of PBMC consisted of CD4 T cells with high CD25 expression, with FoxP3 cells increasing from 5 to 8.5%. These results suggest that ASCs can stimulate the activation and proliferation of Treg-type cells. Treg could be associated with tumor promotion or tumor inhibition, depending on the context. For example, another study reported the increase in Treg cells when ASCs isolated from breast cancer tissue were used in coculture with PBMC lymphocytes [78], and the effect was promotion of what appeared to indicate an anti-inflammatory reaction within breast tumors based on cytokine expression (IL-4, IL-10, TGF*β*, CD25, and CCR4). It remains to be seen whether the net effect would have been tumor suppression or promotion, but the authors suggested that the likely effect would be tumor promotion. Other anti-inflammatory effects were observed in a clinical study where inflammatory nasal polyps (sometimes precancerous) were treated with ASCs. In ASC-treated patients, the proportions of CD4 and CD8 T cells decreased, with reductions in levels of Th2-type cytokines (IL-4 and IL-5) and significant increases in levels of Th1 cytokines (IFN*γ* and IL-2), as well as of regulatory cytokines (TGF*β* and IL-10) [79]. Also, ASCs appeared to have immune regulatory effects by reducing the eosinophilic inflammation of nasal polyps. Downregulation of activated T lymphocytes and a Th2 immune response and upregulation of a Th1 and eosinophilic inflammation could prevent progression of those nasal polyps predisposed to becoming tumors.

**Figure 6:**
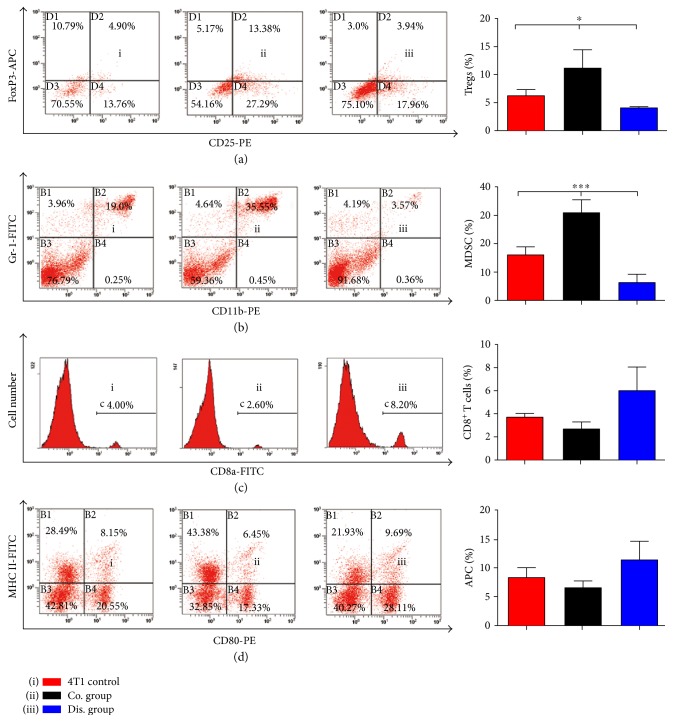
Flow cytometry shows changes in the proportion of immune cells in the spleen of mice on day 30 postinoculation. (a) Change in the proportion of Tregs (CD4^+^CD25^+^Foxp3^+^) of CD4^+^ T cells when gated for positive CD4-FITC cells (*n* = 3; one-way analysis of variance [ANOVA]). Proportions of MDSCs (CD11b^+^Gr-1^+^; *n* = 3; one-way ANOVA) (b), CD8^+^ T cells (*n* = 3; one-way ANOVA) (c), and APCs (CD80^+^MHC II^+^; *n* = 3; one-way ANOVA) (d) were also altered. ^∗^*p* < .05 and ^∗∗∗^*p* < .001.

A provocative new idea to explain the dichotomy between the pro- and antitumor effects of MSCs may depend not only on how they recruit components of the immune system but on their localization within the body when the tumor first starts to develop. A recent study showed opposite effects on breast tumor growth when MSCs were coinjected or injected distantly [80]. Interestingly, in a 4T1 model of breast tumor development, the only variation was the site of injection of MSCs, demonstrating opposite effects on tumor growth for the first time in the same animal model (Figure 6). When injected locally with 4T1 tumor cells (coinjection), MSCs could initially promote the migration and invasion abilities of tumor cells but no significant difference was observed in late-stage lung metastasis. Coinjection of MSCs promoted angiogenesis by participating in the establishment of the tumor stroma. The distant injection of MSCs resulted in tumor-specific migration, presumably to provide structural and functional support to the tumor via differentiation into fibroblastic-like cells and pericytes. However, distant injection inhibited tumor progression and appeared to be directly related to promoting altered immune cell populations within the tumor. Treg and myeloid-derived suppressor cells (MDSC) were decreased significantly; CD8 T cells and APC were increased as a trend (although not significantly). Gene expression profiles of immune cells in the spleen and cytokine analyses of serum suggested that upregulation of TNF, IFN*γ*, TLR3, and IL-12 might explain the antitumor activity of distantly injected MSCs [80]. These interesting findings suggest that naïve MSCs are a double-edged sword in the modulation of tumor growth. In order to harness the potential of MSCs, several groups have genetically modified these cells with the goal of assessing their potential therapeutic effectiveness for a variety of cancers.

Tumor microenvironments are very similar to active sites of chronic inflammation. Since MSCs are able to home to inflammatory sites, researchers explored the possibility of using genetically engineered MSCs as delivery vehicles for antitumor therapies. The first studies utilized MSC engineered to express anticancer genes such as IFN*β*, showing that MSC can engraft and release their therapeutic products within the tumor microenvironment. MSCs that were gene-modified to deliver the immune-stimulating factor (LIGHT), a member of the TNF superfamily, also could induce breast tumor regression *in vivo* [9]. MSC-LIGHT retained tropism towards tumors and stimulated a potent antitumor response that promoted an influx of T cells into tumors and inhibited tumor growth. CD4 T cells were found to play a role in the induction phase of the immune response, and CD8 T cells were essential for the effector phase.

Gene-modified ASCs also can display antitumor activity *in vivo*, and this ability has been shown for a variety of gene products. For example, ASCs stably modified to express IFN*γ* promote significant antimelanoma effects as compared to recombinant IFN*γ* treatment alone [81], suggesting that the mode of delivery of an antitumor cytokine in the tumor microenvironment is critical to its effectiveness. Also, this study showed that ASCs have immune modulatory properties that enhance the effects of IFN*γ* in the tumor microenvironment. ASC-IFN*γ* engrafted into the tumor stroma inhibited tumor growth and angiogenesis, prevented a systemic increase of Treg, increased CD8^+^ T cell infiltration (but not IL-2^+^ cells), and prolonged the survival of mice. A study by our group with ASC stably expressing the glycoprotein and antiangiogenic protein pigment epithelial-derived factor (PEDF) also has shown strong antitumor effects *in vivo* [82]. Like the IFN*γ* study, our group also observed that delivery of molecules secreted by gene-modified ASCs to tumors appears to be more potent than delivering recombinant proteins, as in the case of PEDF. These are interesting observations, but it remains unknown why molecules secreted from ASC are more potent in their antitumor activity—perhaps this can be attributed to the combination of cytokine production with a high production of extravesicles (exosomes) by ASCs. Exosomes bud from the cell to carry mediators which include proteins, miRNA, and mRNA [51], conveying regenerative signals during normal homeostasis, as well as relaying immune modulatory and therapeutic signals during tissue damage between tumor and stromal cells. The role of MSC-derived exosomes is being heavily investigated for therapeutic applications and holds promise for cancer therapy.

#### 3.2.2. Immune-Suppressive Effects of MSCs on Adaptive Immune Cells within the Tumor Microenvironment

The effect of MSC on immune suppression is also well defined in the literature. These effects emerge from cell-cell interactions between MSCs, including ASCs, and both innate and adaptive immune cells [83–85] and are partly mediated through TLR pathways typically through inhibition of T cell proliferation [86]. TLR4 activation has immune-suppressive effects involving vascular cell adhesion molecule 1- and intercellular adhesion molecule 1-mediated binding of immune cells and TLR3 activation via hyaluronic acid interactions [54, 87]. MSC immune-suppressive abilities also can be mediated by the release of soluble factors with anti-inflammatory effects, including TGF*β*, IDO, inducible nitric oxide synthase (iNOS), PGE2, and G-CSF [10]. MSCs also can prevent autoimmunity, as seen in a CCL2-dependent recruitment of myeloid-derived suppressor cells (MDSC), in a mouse model of experimental autoimmune uveitis [88]. Several reports point to a role for ASCs in inducing Treg cells, including those under lower oxygen (5% O_2_) conditions, and other physiologically relevant mechanisms thought to involve cell-cell contact [89].

An alternative source of MSCs is perirenal ASCs, and these have been shown to enhance the percentage of induced Treg cells (iTreg) from effector cells through methylation of a region of the FoxP3 gene promoter [90]. iTreg had immunosuppressive capacities comparable to those of natural Treg (nTreg), and their induction was IL-2 receptor-dependent. The mechanisms employed by MSCs to inhibit effector T cell proliferation seem to overlap with the mechanisms involved in Treg induction, yet they do not interfere with this cell type's function. The inflammatory state also influences which types of chemokines ASCs express, potentially influencing the types of immune cells recruited to the tumor microenvironment. A study examined a coculture of ASCs with alloactivated PBMC in MLR, with proinflammatory cytokines IFN*γ*, TNF*α*, and IL-6 or under control conditions. In the presence of proinflammatory cytokines, ASC upregulated (by >200-fold) the expression of T lymphocyte attractants C-X-C chemokine ligand motif- (CXCL) 9, CXCL-10, and CXCL-11 and also upregulated the neutrophil, monocyte, and eosinophil attractants CXCL1 and CXCL6 (by >7-fold). The pattern of chemokine induction by ASC appeared to depend on the inflammatory stimulus. In ASC cultured with MLR, the expression of CCL-2, CCL-5, CCL-13, CCL-20, and CCL-28 was increased significantly compared to that in control ASC. Culture of ASC with proinflammatory cytokines strongly increased the expression of CCL-2, CCL-5, CCL-7, CCL-8, and CCL-13 but had no effect on the lymphocyte attractants CCL-20 and CCL-28 [91]. Thus, ASCs can be altered by different inflammatory conditions and, importantly, can be preconditioned *in vitro* for potential clinical immune therapy use.

Interestingly, although the research on MSCs has mainly focused on their effects on T cells [17] with data regarding the modulatory effects of MSCs on alloantigen-specific humoral response in humans being scarce, it has been demonstrated recently that MSCs significantly affect B cell functioning [92]. ASCs support the survival of quiescent B cells and target B cell differentiation towards B regulatory cells (Breg, CD19^+^CD24^hi^ CD38^hi^). Such an effect could impact B cell responses in immune diseases such as rheumatoid arthritis, but the effect in the solid tumor microenvironment is currently unknown. The effect on blood tumors such as chronic lymphocytic leukemia (CLL) appears to be bidirectional activation between bone marrow MSC and tumorigenic B cells. Coculture of MSCs protected CLL B cells from both spontaneous and drug-induced apoptosis [1]. The CD38 expression was upregulated in CLL B cells with MSC coculture. In MSC, ERK phosphorylation and AKT phosphorylation were detected when CLL B cells and MSC were separated by a transwell, indicating soluble factor activation of MSC. This study adds to the evidence that in human tumors, including hematological malignancies, stromal cell interaction with tumor cells significantly impacts the critical features of both cell types.

## 4. Polarization of MSC Can Help Explain Their Dual Effect on Tumorigenesis and Immune Modulation

There is increasing evidence that the activity of human MSCs is greatly modulated by the stimulation of TLRs. TLRs are a family of pattern recognition receptors that act upon recognition of pathogen-associated molecular patterns. These triggers act to promote intracellular signaling mechanisms leading to the synthesis and secretion of cytokines by leukocyte subsets and nonimmune cells. Eleven TLRs (TLR1–11) have been identified so far in human cells [93]. Several of these TLRs have been reported to be expressed by MSCs, at different levels depending on the tissue of origin. BM-MSCs have been reported to express TLR1-2 [94–98], TLR3 [86, 94–99], TLR4 [66, 86, 94–99], TLR5-6 [95–99], TLR7 [94–96], TLR9 [94–97], and TLR10 [97]. Wharton jelly-derived MSCs have been reported to express a similar TLR profile than BM-MSCs with the notable absence of TLR4 [95]. And ASCs have been reported consistently to express TLR1–6 and TLR9 [95, 97, 100] and less consistently to express TLR7 [100] and TLR10 [97, 100]. These receptors have been associated with the modulation of multiple MSC properties, including differentiation capability [86], migration [86, 94], extracellular matrix deposition [86], secretome [3, 66, 86, 94–101], immunomodulation [3, 86, 94, 102], and modulation of tumor progression [66, 101]. However, inconsistent reports of the modulation of these properties are found in the literature. As we have discussed in previous sections, the role of MSCs in immunomodulation is primarily achieved by the secretion of cytokines that affect the activity of immune cells. Thus, multiple groups have examined the role of changes in the secretome of MSCs upon TLR ligation (primarily TLR3 and TLR4) in order to understand the role of TLR2 in MSC-mediated immunomodulation.

Multiple studies report similar responses from the stimulation of TLR3 or TLR4 on BM-MSCs and ASCs with their respective agonists LPS and polyinosinic:polycytidylic acid (poly(I:C)). Stimulation with these ligands has been reported to promote the expression of cytokines and chemokines with roles in immunomodulation and inflammation such as CXCL10 [94], IL-6 [86, 94–97, 100, 101], IL-8 [86, 94–96, 100], CCL5 [95, 96], IL-12 [94, 95, 97], IL-27 [95], IL-23 [95], IL-1*β* [96], MIP3*α* [97], TNF*α* [94, 97], and CCL2 [97]. Similarly, TLR ligation with poly(I:C) and LPS has been associated with activation of NF*κ*B signaling [100]. Although many of the upregulated cytokines have roles in the modulation of immune cells, including neutrophils, lymphocytes, DCs, macrophages, and NK cells, there is little to no consensus on the effect of TLR ligation on immunomodulation. In 2009, Lombardo et al. concluded that TLR ligation did not have a significant effect on the immunogenic properties of hASCs when they evaluated the immune-modulating activity on peripheral blood lymphocyte proliferation or activation [100]. In contrast, Cassatella et al. and Liotta et al. reported a reduction in the immunosuppressive activity of MSCs that promoted the T cell and neutrophil survival, activation, and response upon TLR ligation [54, 102].

**Figure 7:**
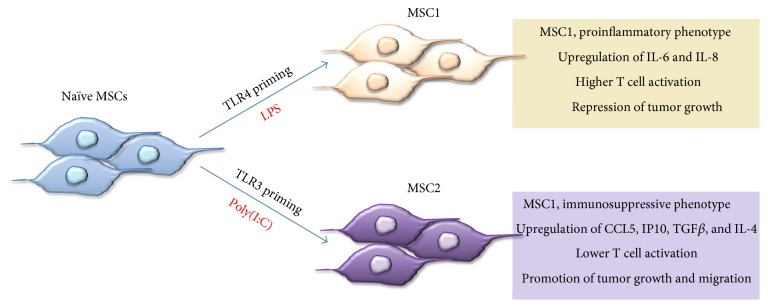
Interactions of MSC with the microenvironment leading to polarization. MSC polarization by different stimuli such as LPS or poly(I:C) through stimulation of either TLR4 or TLR3 receptors, respectively. Two polarized MSC phenotypes emerge from TLR stimulation, which represent a proinflammatory phenotype (MSC1, antitumorigenic) and immune-suppressive phenotype (MSC2, protumorigenic).

Although most studies report similar results following stimulation with poly(I:C) or LPS, contrasting phenotypes have been described for the stimulation of these TLRs in BM-MSCs. These phenotypes were described as the proinflammatory MSC1 and the immunosuppressive MSC2 [86] (Figure 7). Characterization of these phenotypes led to the understanding that the low-level exposure of MSCs to the TLR4 ligand generates the MSC1 phenotype, whereas the ligation of TLR3 to double-stranded RNA or poly(I:C) generates the MSC2 phenotype. MSC1 shows an increased synthesis and secretion of proinflammatory cytokines and chemokines, such as IL-6 and IL-8, whereas MSC2 has increased production of immunosuppressive mediators such as IP-10 and CCL5. Other phenotypes are polarized as well. For instance, MSC1 has been reported to permit T lymphocyte activation and attenuate the tumor progression relative to naïve MSCs. On the contrary, MSC2 has been associated with the suppression of T lymphocytes and the promotion of tumor growth and metastasis [3, 86]. Multiple factors account for the variability of responses seen upon the stimulation of TLRs in MSCs, including tissue of origin [95], species of origin, and environmental conditions [97, 98].

Exposure to inflammatory cytokines has been reported to alter the TLR profile of MSCs [96, 98], causing an upregulation of TLR2-3 [95, 96, 98], TLR4 [95, 98], and TLR7 [96] and a downregulation of TLR6 [98] which causes a change in the responsiveness of the cells to TLR stimulation [98]. Additionally, Romieu-Mourez et al. reported that the combination of TLR3 and TLR4 stimulation with inflammatory cytokines increases the response of the MSCs and can synergistically upregulate the secretion of certain cytokines and enzymes [96]. IFN*α* and poly(I:C) synergistically upregulated IL-12, TNF*α*, CCL5, IFN*β*, and iNOS. Similarly, the two ligands acted in synergy with IFN*γ* for the upregulation of CCL5, TRAIL, TNF*α*, IL-12, and iNOS. Additionally, increased neutrophil chemotaxis was observed when attracting the cells with conditioned media from hMSCs treated with IFN*γ* and LPS. This increased chemotaxis was associated with the increased secretion of IL-6 and IL-8 resulting from this combined treatment.

In 2015, tumor-derived MSCs from acute myeloid leukemia and lung cancer tissues and cells cultured in conditioned media from HeLa cells were reported to present a higher expression of TLR4 compared to unsorted MSCs [66]. These MSCs were also found to have a lower secretion of IL-6 and IL-8 than other MSCs. However, the secretion of these cytokines experiences a more significant expression enhancement upon LPS stimulation. Additionally, NK cell proliferation was suppressed by these TLR4^+^ MSC, and the suppression was enhanced by the activation of the cells with LPS.

The study of TLR ligation effects on MSCs could provide key information relevant to the development of treatments for the targeting of different types of cancer [103] and inflammatory diseases [104–106]. However, the current understanding of these phenomena is challenging due to the variability in stimulation conditions (i.e., ligand concentration, base media, time of exposure), sources (i.e., donor, tissue of origin, species), and culture conditions (i.e., media passaging) of cells among the multiple studies, resulting in the inconsistent phenotypes currently found in the literature.

## 5. Harnessing the Power of MSC for Immune Therapy

Understanding and harnessing the polarization of MSCs in the tumor might provide us with tools to augment effectiveness of current and future immune therapies. Eight current or recent clinical studies point to the promise of using MSCs (naïve or gene-modified) for cancer therapy. These studies include several phase I trials including MSC expressing IFN*γ* or MSC bearing the sodium symporter gene for ovarian cancer therapy, as well as allogeneic BM-MSC for localized prostate cancer, and MSC bearing GX-051 for advanced head and neck cancer. Phase 1-2 trials are being held for MSC coinfused with hematopoietic stem cells for treating leukemia and other myeloproliferative disorders and MSC bearing CRAd (oncolytic adenoviruses) for children and adults with multiple types of metastatic and refractory solid tumors.

Additionally, since MSCs (and ASC) are found in stromal cell niches of various tumor types, it is possible that MSC can exploit the properties related to tissue repair to promote tumorigenesis and/or protect epithelial cells from the effects of chemotherapy. By restoring the MSC ability to modulate anticancer immunity, perhaps one can hijack the tumor to favor infiltration of immune cells and reduce tumor bulk or reduce metastasis.

## 6. Conclusions

MSC biology exhibits high plasticity and thus should be studied in a relevant environmental context. The several studies we have reviewed here only begin to shed light on the effects of each variable in the microenvironment on tumor progression, as each variable is usually tested and reported in isolation. In reality, MSCs either are in their natural environment or are homing to new environments to encounter various signals at the same time and in various sequences. The complexity of tumor microenvironments, for example, makes the interpretation of studies that focus on a few molecules difficult, and it is important to remember to draw limited conclusions about each study. The same principle goes for tumor-derived MSCs or stromal cells being used to deliver anticancer therapy or modulate cancer immunity. Once MSCs arrive at the cancer site, they meet epithelial tumor cells and stromal cell-derived factors in high concentrations, including cytokines, chemokines, other immunomodulatory small molecules, and various DAMPs coming from dying malignant cells. Ultimately, the result of all these environmental factors will determine how MSCs will actually behave *in vivo*. Different cells contribute to tumor development, including the classically described tumor stromal cell components and also MSC in the local tumor stroma, interconnected in a network of crosstalk and mutual modulation. The network includes cell-cell contact and a specific secretory profile acquired by MSCs during this interaction, enabling them to perform as proinflammatory cells or as anti-inflammatory cells and to alter the tumor microenvironment. The application of MSC for cell therapy purposes will have several benefits over that of other stem cells including induced pluripotent stem cells including the possibility of easy availability, low in vitro manipulation requirements, potential autologous application, and a lower risk of tumorigenicity. MSCs have high plasticity in adapting to different tumor microenvironments, raising the possibility of experimental modulation or priming of their “phenotype.” The therapeutic potential of MSCs offers enormous hope for treating tissue defects and numerous diseases, including cancer.

## 7. Future Directions

MSCs can self-renew, differentiate into multiple lineages, and exhibit proangiogenic and immunomodulatory effects. Along with these intrinsic properties, MSCs exhibit natural tropism toward inflamed tissue, which has led to the clinical application of these cells in different therapies. However, a significant barrier is the inability to localize the cells to the tissue of interest due to low homing efficiency, poor engraftment, and low cell retention. To circumvent these challenges, it is critical to develop engineering strategies that can improve tissue engraftment as well as enhance the therapeutic potential of these cells. The therapeutic application of MSC (or ASC) will require a relatively long-term culturing method, which can result in senescence of cells and a potential reduction in the therapeutic activity of transplanted cells. If the immune-suppressive or immune-stimulatory capacity of MSC can be restored via careful and purposeful “polarization,” their application might be harnessed to its full therapeutic potential.

## References

[1] Melzer C., Yang Y., Hass R. (2016). Interaction of MSC with tumor cells. *Cell Communication and Signaling*.

[2] Horwitz E. M., Le Blanc K., Dominici M. (2005). Clarification of the nomenclature for MSC: the International Society for Cellular Therapy position statement. *Cytotherapy*.

[3] Waterman R. S., Henkle S. L., Betancourt A. M. (2012). Mesenchymal stem cell 1 (MSC1)-based therapy attenuates tumor growth whereas MSC2-treatment promotes tumor growth and metastasis. *PLoS One*.

[4] Klyushnenkova E., Mosca J. D., Zernetkina V. (2005). T cell responses to allogeneic human mesenchymal stem cells: immunogenicity, tolerance, and suppression. *Journal of Biomedical Science*.

[5] Dominici M., Le Blanc K., Mueller I. (2006). Minimal criteria for defining multipotent mesenchymal stromal cells. The International Society for Cellular Therapy position statement. *Cytotherapy*.

[6] Keating A. (2012). Mesenchymal stromal cells: new directions. *Cell Stem Cell*.

[7] Keating A. (2006). Mesenchymal stromal cells. *Current Opinion in Hematology*.

[8] Pittenger M. F., Mackay A. M., Beck S. C. (1999). Multilineage potential of adult human mesenchymal stem cells. *Science*.

[9] Horwitz E. M., Andreef M., Frassoni F. (2006). Mesenchymal stromal cells. *Current Opinion in Hematology*.

[10] Nwabo Kamdje A., Vecchio L., Seke Etet P. F. (2016). Mesenchymal stem/stromal cell trafficking and homing. *Mesenchymal Stromal Cells as Tumor Stromal Modulators*.

[11] Meirelles Lda S., Fontes A. M., Covas D. T., Caplan A. I. (2009). Mechanisms involved in the therapeutic properties of mesenchymal stem cells. *Cytokine & Growth Factor Reviews*.

[12] Zimmerlin L., Park T. S., Zambidis E. T., Donnenberg V. S., Donnenberg A. D. (2013). Mesenchymal stem cell secretome and regenerative therapy after cancer. *Biochimie*.

[13] Kidd S., Spaeth E., Watson K. (2012). Origins of the tumor microenvironment: quantitative assessment of adipose-derived and bone marrow-derived stroma. *PLoS One*.

[14] Bouchlaka M., Hematti P., Capitini C. M., Bolontrade M., Garcia M. (2016). Therapeutic purposes and risks of ex vivo expanded mesenchymal stem/stromal cells. *Mesenchymal Stromal Cells as Tumor Stromal Modulators*.

[15] Walker N. D., Nahas G. R., Munoz J., Lucas J., Pobiarzyn P., Rameshwar P. (2016). Mesenchymal stem/stromal cells and the tumor immune system. *Mesenchymal Stromal Cells as Tumor Stromal Modulators*.

[16] Fotia C., Massa A., Boriani F., Baldini N., Granchi D. (2015). Prolonged exposure to hypoxic milieu improves the osteogenic potential of adipose derived stem cells. *Journal of Cellular Biochemistry*.

[17] Zimmerlin L., Donnenberg A. D., Rubin J. P., Basse P., Landreneau R. J., Donnenberg V. S. (2011). Regenerative therapy and cancer: in vitro and in vivo studies of the interaction between adipose-derived stem cells and breast cancer cells from clinical isolates. *Tissue Engineering Part A*.

[18] Rhee I. (2016). Diverse macrophages polarization in tumor microenvironment. *Archives of Pharmacal Research*.

[19] Casazza A., Di Conza G., Wenes M., Finisguerra V., Deschoemaeker S., Mazzone M. (2014). Tumor stroma: a complexity dictated by the hypoxic tumor microenvironment. *Oncogene*.

[20] Parmar K., Mauch P., Vergilio J. A., Sackstein R., Down J. D. (2007). Distribution of hematopoietic stem cells in the bone marrow according to regional hypoxia. *Proceedings of the National Academy of Sciences of the United States of America*.

[21] Fotia C., Massa A., Boriani F., Baldini N., Granchi D. (2015). Hypoxia enhances proliferation and stemness of human adipose-derived mesenchymal stem cells. *Cytotechnology*.

[22] Fehrer C., Brunauer R., Laschober G. (2007). Reduced oxygen tension attenuates differentiation capacity of human mesenchymal stem cells and prolongs their lifespan. *Aging Cell*.

[23] Hung S. P., Ho J. H., Shih Y. R., Lo T., Lee O. K. (2012). Hypoxia promotes proliferation and osteogenic differentiation potentials of human mesenchymal stem cells. *Journal of Orthopaedic Research*.

[24] Zhou N., Hu N., Liao J. Y. (2015). HIF-1α as a regulator of BMP2-induced chondrogenic differentiation, osteogenic differentiation, and endochondral ossification in stem cells. *Cellular Physiology and Biochemistry*.

[25] Paquet J., Deschepper M., Moya A., Logeart-Avramoglou D., Boisson-Vidal C., Petite H. (2015). Oxygen tension regulates human mesenchymal stem cell paracrine functions. *Stem Cells Translational Medicine*.

[26] Buravkova L. B., Andreeva E. R., Gogvadze V., Zhivotovsky B. (2014). Mesenchymal stem cells and hypoxia: where are we?. *Mitochondrion*.

[27] Ejtehadifar M., Shamsasenjan K., Movassaghpour A. (2015). The effect of hypoxia on mesenchymal stem cell biology. *Advanced Pharmaceutical Bulletin*.

[28] Madrigal M., Rao K. S., Riordan N. H. (2014). A review of therapeutic effects of mesenchymal stem cell secretions and induction of secretory modification by different culture methods. *Journal of Translational Medicine*.

[29] Valorani M. G., Montelatici E., Germani A. (2012). Pre-culturing human adipose tissue mesenchymal stem cells under hypoxia increases their adipogenic and osteogenic differentiation potentials. *Cell Proliferation*.

[30] Basciano L., Nemos C., Foliguet B. (2011). Long term culture of mesenchymal stem cells in hypoxia promotes a genetic program maintaining their undifferentiated and multipotent status. *BMC Cell Biology*.

[31] Kim D. S., Ko Y. J., Lee M. W. (2016). Effect of low oxygen tension on the biological characteristics of human bone marrow mesenchymal stem cells. *Cell Stress and Chaperones*.

[32] Chaturvedi P., Gilkes D. M., Takano N., Semenza G. L. (2014). Hypoxia-inducible factor-dependent signaling between triple-negative breast cancer cells and mesenchymal stem cells promotes macrophage recruitment. *Proceedings of the National Academy of Sciences of the United States of America*.

[33] Han K. H., Kim A. K., Kim M. H., Kim D. H., Go H. N., Kim D. I. (2016). Enhancement of angiogenic effects by hypoxia-preconditioned human umbilical cord-derived mesenchymal stem cells in a mouse model of hindlimb ischemia. *Cell Biology International*.

[34] Lakatos K., Kalomoiris S., Merkely B., Nolta J. A., Fierro F. A. (2016). Mesenchymal stem cells respond to hypoxia by increasing diacylglycerols. *Journal of Cellular Biochemistry*.

[35] Liu J., Hao H., Xia L. (2015). Hypoxia pretreatment of bone marrow mesenchymal stem cells facilitates angiogenesis by improving the function of endothelial cells in diabetic rats with lower ischemia. *PLoS One*.

[36] Wong C. C., Gilkes D. M., Zhang H. (2011). Hypoxia-inducible factor 1 is a master regulator of breast cancer metastatic niche formation. *Proceedings of the National Academy of Sciences of the United States of America*.

[37] Kavanagh D. P., Suresh S., Newsome P. N., Frampton J., Kalia N. (2015). Pretreatment of mesenchymal stem cells manipulates their vasculoprotective potential while not altering their homing within the injured gut. *Stem Cells*.

[38] Németh K., Leelahavanichkul A., Yuen P. S. (2009). Bone marrow stromal cells attenuate sepsis via prostaglandin E_2_-dependent reprogramming of host macrophages to increase their interleukin-10 production. *Nature Medicine*.

[39] Danchuk S., Ylostalo J. H., Hossain F. (2011). Human multipotent stromal cells attenuate lipopolysaccharide-induced acute lung injury in mice via secretion of tumor necrosis factor-α-induced protein 6. *Stem Cell Research & Therapy*.

[40] Cao W., Cao K., Cao J., Wang Y., Shi Y. (2015). Mesenchymal stem cells and adaptive immune responses. *Immunology Letters*.

[41] Ren G., Zhao X., Zhang L. (2010). Inflammatory cytokine-induced intercellular adhesion molecule-1 and vascular cell adhesion molecule-1 in mesenchymal stem cells are critical for immunosuppression. *The Journal of Immunology*.

[42] Sheng H., Wang Y., Jin Y. (2008). A critical role of IFNγ in priming MSC-mediated suppression of T cell proliferation through up-regulation of B7-H1. *Cell Research*.

[43] Chan J. L., Tang K. C., Patel A. P. (2006). Antigen-presenting property of mesenchymal stem cells occurs during a narrow window at low levels of interferon-γ. *Blood*.

[44] Chan W. K., Lau A. S., Li J. C., Law H. K., Lau Y. L., Chan G. C. (2008). MHC expression kinetics and immunogenicity of mesenchymal stromal cells after short-term IFN-γ challenge. *Experimental Hematology*.

[45] Roelen B. A., Dijke P. (2003). Controlling mesenchymal stem cell differentiation by TGFβ family members. *Journal of Orthopaedic Science*.

[46] Tang D., Kang R., Coyne C. B., Zeh H. J., Lotze M. T. (2012). PAMPs and DAMPs: signal 0s that spur autophagy and immunity. *Immunological Reviews*.

[47] Eisenbacher J. L., Schrezenmeier H., Jahrsdörfer B. (2014). S100A4 and uric acid promote mesenchymal stromal cell induction of IL-10^+^/IDO^+^ lymphocytes. *The Journal of Immunology*.

[48] Yañez R., Oviedo A., Aldea M., Bueren J. A., Lamana M. L. (2010). Prostaglandin E2 plays a key role in the immunosuppressive properties of adipose and bone marrow tissue-derived mesenchymal stromal cells. *Experimental Cell Research*.

[49] Droujinine I. A., Eckert M. A., Zhao W. (2013). To grab the stroma by the horns: from biology to cancer therapy with mesenchymal stem cells. *Oncotarget*.

[50] Lin W. W., Karin M. (2007). A cytokine-mediated link between innate immunity, inflammation, and cancer. *The Journal of Clinical Investigation*.

[51] Gudbergsson J. M., Duroux M. (2016). Extracellular vesicles from mesenchymal stem cells and their potential in tumor therapy. *Mesenchymal Stromal Cells as Tumor Stromal Modulators*.

[52] Glenn J. D., Whartenby K. A. (2014). Mesenchymal stem cells: emerging mechanisms of immunomodulation and therapy. *World Journal of Stem Cells*.

[53] Kim J., Hematti P. (2009). Mesenchymal stem cell-educated macrophages: a novel type of alternatively activated macrophages. *Experimental Hematology*.

[54] Cassatella M. A., Mosna F., Micheletti A. (2011). Toll-like receptor-3-activated human mesenchymal stromal cells significantly prolong the survival and function of neutrophils. *Stem Cells*.

[55] Hall S. R., Tsoyi K., Ith B. (2013). Mesenchymal stromal cells improve survival during sepsis in the absence of heme oxygenase-1: the importance of neutrophils. *Stem Cells*.

[56] Jaiswal S., Chao M. P., Majeti R., Weissman I. L. (2010). Macrophages as mediators of tumor immunosurveillance. *Trends in Immunology*.

[57] Jia X. H., Feng G. W., Wang Z. L. (2016). Activation of mesenchymal stem cells by macrophages promotes tumor progression through immune suppressive effects. *Oncotarget*.

[58] Kudlik G., Hegyi B., Czibula Á., Monostori É., Buday L., Uher F. (2016). Mesenchymal stem cells promote macrophage polarization toward M2b-like cells. *Experimental Cell Research*.

[59] Wolfe A. R., Trenton N. J., Debeb B. G. (2016). Mesenchymal stem cells and macrophages interact through IL-6 to promote inflammatory breast cancer in pre-clinical models. *Oncotarget*.

[60] Fisher D. T., Appenheimer M. M., Evans S. S. (2014). The two faces of IL-6 in the tumor microenvironment. *Seminars in Immunology*.

[61] Mathew E., Brannon A. L., Del Vecchio A. (2016). Mesenchymal stem cells promote pancreatic tumor growth by inducing alternative polarization of macrophages. *Neoplasia*.

[62] Vivier E., Ugolini S., Blaise D., Chabannon C., Brossay L. (2012). Targeting natural killer cells and natural killer T cells in cancer. *Nature Reviews Immunology*.

[63] Poli A., Michel T., Thérésine M., Andrès E., Hentges F., Zimmer J. (2009). CD56^bright^ natural killer (NK) cells: an important NK cell subset. *Immunology*.

[64] Ribeiro A., Laranjeira P., Mendes S. (2013). Mesenchymal stem cells from umbilical cord matrix, adipose tissue and bone marrow exhibit different capability to suppress peripheral blood B, natural killer and T cells. *Stem Cell Research & Therapy*.

[65] Liu W., Gao Y., Li H. (2016). Intravenous transplantation of mesenchymal stromal cells has therapeutic effects in a sepsis mouse model through inhibition of septic natural killer cells. *The International Journal of Biochemistry & Cell Biology*.

[66] Lu Y., Liu J., Liu Y. (2015). TLR4 plays a crucial role in MSC-induced inhibition of NK cell function. *Biochemical and Biophysical Research Communications*.

[67] Entrena A., Varas A., Vázquez M. (2015). Mesenchymal stem cells derived from low risk acute lymphoblastic leukemia patients promote NK cell antitumor activity. *Cancer Letters*.

[68] Yu Y., Zhang Q., Meng Q. (2016). Mesenchymal stem cells overexpressing Sirt1 inhibit prostate cancer growth by recruiting natural killer cells and macrophages. *Oncotarget*.

[69] Ma Y., Shurin G. V., Peiyuan Z., Shurin M. R. (2013). Dendritic cells in the cancer microenvironment. *Journal of Cancer*.

[70] Liu W. H., Liu J. J., Wu J. (2013). Novel mechanism of inhibition of dendritic cells maturation by mesenchymal stem cells via interleukin-10 and the JAK1/STAT3 signaling pathway. *PLoS One*.

[71] Abomaray F. M., Al Jumah M. A., Kalionis B. (2015). Human chorionic villous mesenchymal stem cells modify the functions of human dendritic cells, and induce an anti-inflammatory phenotype in CD_1+_ dendritic cells. *Stem Cell Reviews*.

[72] Ghosh T., Barik S., Bhuniya A. (2016). Tumor-associated mesenchymal stem cells inhibit naïve T cell expansion by blocking cysteine export from dendritic cells. *International Journal of Cancer*.

[73] Zhao Z. G., Xu W., Sun L., Li W. M., Li Q. B., Zou P. (2012). The characteristics and immunoregulatory functions of regulatory dendritic cells induced by mesenchymal stem cells derived from bone marrow of patient with chronic myeloid leukaemia. *European Journal of Cancer*.

[74] Mougiakakos D., Choudhury A., Lladser A., Kiessling R., Johansson C. C. (2010). Regulatory T cells in cancer. *Advances in Cancer Research*.

[75] Crop M. J., Baan C. C., Korevaar S. S., Ijzermans J. N., Weimar W., Hoogduijn M. J. (2010). Human adipose tissue-derived mesenchymal stem cells induce explosive T-cell proliferation. *Stem Cells and Development*.

[76] Stagg J., Pommey S., Eliopoulos N., Galipeau J. (2006). Interferon-γ-stimulated marrow stromal cells: a new type of nonhematopoietic antigen-presenting cell. *Blood*.

[77] Ma S., Xie N., Li W., Yuan B., Shi Y., Wang Y. (2014). Immunobiology of mesenchymal stem cells. *Cell Death and Differentiation*.

[78] Razmkhah M., Jaberipour M., Erfani N., Habibagahi M., Talei A. R., Ghaderi A. (2011). Adipose derived stem cells (ASCs) isolated from breast cancer tissue express IL-4, IL-10 and TGF-*β*1 and upregulate expression of regulatory molecules on T cells: do they protect breast cancer cells from the immune response?. *Cellular Immunology*.

[79] Cho K. S., Kim Y. W., Kang M. J., Park H. Y., Hong S. L., Roh H. J. (2014). Immunomodulatory effect of mesenchymal stem cells on T lymphocyte and cytokine expression in nasal polyps. *Otolaryngology-Head and Neck Surgery*.

[80] Zheng H., Zou W., Shen J. (2016). Opposite effects of coinjection and distant injection of mesenchymal stem cells on breast tumor cell growth. *Stem Cells Translational Medicine*.

[81] Bahrambeigi V., Ahmadi N., Moisyadi S., Urschitz J., Salehi R., Haghjooy Javanmard S. (2014). PhiC31/PiggyBac modified stromal stem cells: effect of interferon γ and/or tumor necrosis factor (TNF)-related apoptosis-inducing ligand (TRAIL) on murine melanoma. *Molecular Cancer*.

[82] Zolochevska O., Yu G., Gimble J. M., Figueiredo M. L. (2012). Pigment epithelial-derived factor and melanoma differentiation associated gene-7 cytokine gene therapies delivered by adipose-derived stromal/mesenchymal stem cells are effective in reducing prostate cancer cell growth. *Stem Cells and Development*.

[83] Nwabo Kamdje A. H., Mosna F., Bifari F. (2011). Notch-3 and Notch-4 signaling rescue from apoptosis human B-ALL cells in contact with human bone marrow-derived mesenchymal stromal cells. *Blood*.

[84] Calkoen F. G., Brinkman D. M., Vervat C. (2013). Mesenchymal stromal cells isolated from children with systemic juvenile idiopathic arthritis suppress innate and adaptive immune responses. *Cytotherapy*.

[85] Reading J. L., Yang J. H., Sabbah S. (2013). Clinical-grade multipotent adult progenitor cells durably control pathogenic T cell responses in human models of transplantation and autoimmunity. *The Journal of Immunology*.

[86] Waterman R. S., Tomchuck S. L., Henkle S. L., Betancourt A. M. (2010). A new mesenchymal stem cell (MSC) paradigm: polarization into a pro-inflammatory MSC1 or an immunosuppressive MSC2 phenotype. *PLoS One*.

[87] Kota D. J., DiCarlo B., Hetz R. A., Smith P., Cox C. S., Olson S. D. (2014). Differential MSC activation leads to distinct mononuclear leukocyte binding mechanisms. *Scientific Reports*.

[88] Lee H. J., Ko J. H., Jeong H. J. (2015). Mesenchymal stem/stromal cells protect against autoimmunity via CCL2-dependent recruitment of myeloid-derived suppressor cells. *The Journal of Immunology*.

[89] Rowan B. G., Gimble J. M., Sheng M. (2014). Human adipose tissue-derived stromal/stem cells promote migration and early metastasis of triple negative breast cancer xenografts. *PLoS One*.

[90] Engela A. U., Baan C. C., Litjens N. H. (2013). Mesenchymal stem cells control alloreactive CD8^+^ CD28^−^ T cells. *Clinical & Experimental Immunology*.

[91] Crop M. J., Baan C. C., Korevaar S. S. (2010). Inflammatory conditions affect gene expression and function of human adipose tissue-derived mesenchymal stem cells. *Clinical & Experimental Immunology*.

[92] Franquesa M., Mensah F. K., Huizinga R. (2015). Human adipose tissue-derived mesenchymal stem cells abrogate plasmablast formation and induce regulatory B cells independently of T helper cells. *Stem Cells*.

[93] Yu L., Wang L., Chen S. (2010). Endogenous toll-like receptor ligands and their biological significance. *Journal of Cellular and Molecular Medicine*.

[94] Tomchuck S. L., Zwezdaryk K. J., Coffelt S. B., Waterman R. S., Danka E. S., Scandurro A. B. (2008). Toll-like receptors on human mesenchymal stem cells drive their migration and immunomodulating responses. *Stem Cells*.

[95] Raicevic G., Najar M., Stamatopoulos B. (2011). The source of human mesenchymal stromal cells influences their TLR profile as well as their functional properties. *Cellular Immunology*.

[96] Romieu-Mourez R., François M., Boivin M. N., Bouchentouf M., Spaner D. E., Galipeau J. (2009). Cytokine modulation of TLR expression and activation in mesenchymal stromal cells leads to a proinflammatory phenotype. *The Journal of Immunology*.

[97] Hwa Cho H., Bae Y. C., Jung J. S. (2006). Role of toll-like receptors on human adipose-derived stromal cells. *Stem Cells*.

[98] Raicevic G., Rouas R., Najar M. (2010). Inflammation modifies the pattern and the function of Toll-like receptors expressed by human mesenchymal stromal cells. *Human Immunology*.

[99] Chen X., Zhang Z. Y., Zhou H., Zhou G. W. (2014). Characterization of mesenchymal stem cells under the stimulation of Toll-like receptor agonists. *Development, Growth & Differentiation*.

[100] Lombardo E., DelaRosa O., Mancheño-Corvo P., Menta R., Ramírez C., Büscher D. (2009). Toll-like receptor-mediated signaling in human adipose-derived stem cells: implications for immunogenicity and immunosuppressive potential. *Tissue Engineering Part A*.

[101] Pevsner-Fischer M., Morad V., Cohen-Sfady M. (2007). Toll-like receptors and their ligands control mesenchymal stem cell functions. *Blood*.

[102] Liotta F., Angeli R., Cosmi L. (2008). Toll-like receptors 3 and 4 are expressed by human bone marrow-derived mesenchymal stem cells and can inhibit their T-cell modulatory activity by impairing Notch signaling. *Stem Cells*.

[103] Reagan M. R., Kaplan D. L. (2011). Concise review: mesenchymal stem cell tumor-homing: detection methods in disease model systems. *Stem Cells*.

[104] Waterman R. S., Morgenweck J., Nossaman B. D., Scandurro A. E., Scandurro S. A., Betancourt A. M. (2012). Anti-inflammatory mesenchymal stem cells (MSC2) attenuate symptoms of painful diabetic peripheral neuropathy. *Stem Cells Translational Medicine*.

[105] Sangiorgi B., Panepucci R. A. (2016). Modulation of immunoregulatory properties of mesenchymal stromal cells by toll-like receptors: potential applications on GVHD. *Stem Cells International*.

[106] DelaRosa O., Lombardo E. (2010). Modulation of adult mesenchymal stem cells activity by toll-like receptors: implications on therapeutic potential. *Mediators of Inflammation*.

